# Characterization of the Complete Mitochondrial Genome of Eight Diurnal Hawkmoths (Lepidoptera: Sphingidae): New Insights into the Origin and Evolution of Diurnalism in Sphingids

**DOI:** 10.3390/insects13100887

**Published:** 2022-09-29

**Authors:** Yi-Xin Huang, Zhi-Ping Xing, Hao Zhang, Zhen-Bang Xu, Li-Long Tao, Hao-Yuan Hu, Ian J. Kitching, Xu Wang

**Affiliations:** 1 Collaborative Innovation Center of Recovery and Reconstruction of Degraded Ecosystem in Wanjiang Basin Co-Founded by Anhui Province and Ministry of Education, School of Ecology and Environment, Anhui Normal University, Wuhu 241000, China; 2 Key Laboratory of the Zoological Systematics and Evolution, Institute of Zoology, Chinese Academy of Sciences, No. 1 Beichen West Road, Chaoyang District, Beijing 100101, China; 3 Anhui Provincial Key Laboratory of the Conservation and Exploitation of Biological Resources, College of Life Sciences, Anhui Normal University, Wuhu 241000, China; 4 Institute of Resource Plants, Yunnan University, Kunming 650500, China; 5 Natural History Museum, Cromwell Road, London SW7 5BD, UK

**Keywords:** Sphingidae, mitophylogenomics, diurnal behavior, ancestral character reconstruction, divergence time estimation

## Abstract

**Simple Summary:**

We newly sequenced the mitochondrial genomes of 22 species from three subfamilies in the family Sphingidae. The mitogenomes of six diurnal species were newly sequenced, assembled, and annotated in this study (*Hemaris radians*, *Macroglossum bombylans*, *Macroglossum fritzei*, *Macroglossum pyrrhosticta*, *Neogurelca* *himachala*, and *Sataspes xylocoparis*) and, together with those of two previously reported diurnal hawkmoths (*Cephonodes hylas* and *Macroglossum stellatarum*), were analyzed in terms of sequence length, nucleotide composition, relative synonymous codon usage, non-synonymous/synonymous substitution ratio, gene spacing, and repeat sequences. The phylogenetic relationships among the subfamilies of Sphingidae were analyzed to explore the origin, divergence time, and evolutionary status of diurnal sphingids. In addition, we performed divergence time estimation and reconstruction of ancestral characters. Diurnal behavior of hawkmoths first originated 29.19 million years ago (Mya) and may have been influenced by the combination of herbaceous flourishing, which occurred 26–28 Mya, the uplift of the Tibetan Plateau, and the large-scale evolution of bats in the Oligocene to Pre-Miocene. Moreover, diurnalism in hawkmoths had multiple independent origins.

**Abstract:**

In this study, the mitochondrial genomes of 22 species from three subfamilies in the Sphingidae were sequenced, assembled, and annotated. Eight diurnal hawkmoths were included, of which six were newly sequenced (*Hemaris radians*, *Macroglossum bombylans*, *M. fritzei*, *M. pyrrhosticta*, *Neogurelca himachala*, and *Sataspes xylocoparis*) and two were previously published (*Cephonodes hylas* and *Macroglossum stellatarum*). The mitochondrial genomes of these eight diurnal hawkmoths were comparatively analyzed in terms of sequence length, nucleotide composition, relative synonymous codon usage, non-synonymous/synonymous substitution ratio, gene spacing, and repeat sequences. The mitogenomes of the eight species, ranging in length from 15,201 to 15,461 bp, encode the complete set of 37 genes usually found in animal mitogenomes. The base composition of the mitochondrial genomes showed A+T bias. The most commonly used codons were UUA (Leu), AUU (Ile), UUU (Phe), AUA (Met), and AAU (Asn), whereas GCG (Ala) and CCG (Pro) were rarely used. A phylogenetic tree of Sphingidae was constructed based on both maximum likelihood and Bayesian methods. We verified the monophyly of the four current subfamilies of Sphingidae, all of which had high support. In addition, we performed divergence time estimation and ancestral character reconstruction analyses. Diurnal behavior in hawkmoths originated 29.19 million years ago (Mya). It may have been influenced by the combination of herbaceous flourishing, which occurred 26–28 Mya, the uplift of the Tibetan Plateau, and the large-scale evolution of bats in the Oligocene to Pre-Miocene. Moreover, diurnalism in hawkmoths had multiple independent origins in Sphingidae.

## 1. Introduction

Hawkmoths, of the lepidopteran family Sphingidae, are diverse and numerous, with a worldwide distribution except for a few areas with extreme climatic conditions, such as Antarctica and some deserts. They are important subjects of research in physiology, biogeography, molecular systematics, and conservation, as well as agricultural entomology. The larvae are phytophagous, and a number of species feed on important crops, such as cassava, fruit trees, legumes, and other vegetables, and can cause significant losses to agriculture [1]. Therefore, research into the systematics and biology of hawkmoths can help further our understanding of their agroeconomic role and suggest means by which we can reduce the economic losses caused by these insects. Most adult hawkmoths rest during the day, becoming active at dusk and night. However, a minority of species, mostly in the subfamily Macroglossinae, including species in the genera *Cephonodes* Hübner, 1819, *Hemaris* Dalman, 1816, and *Macroglossum* Scopoli, 1777, have a diurnal lifestyle [2]. Current research on diurnal hawkmoths is mainly focused on olfactory and visual physiology [3,4]. In support of such research, comparative studies on the mitochondrial genome of diurnal hawkmoths are important.

Mitochondria are very important organelles in eukaryotic organisms and are present in almost all species. The insect mitochondrial genome is a closed loop of double-stranded DNA, mostly 14–20 kb in length, and the complete mitochondrial genome contains 37 genes, namely 13 PCG, 22 tRNA, and 2 rRNA genes [5]. The mitochondrial genome has different genetic characteristics from the nuclear genome and is often referred to as a second genetic information system [6]. Insect mitochondrial genomes are widely used as molecular markers in population genetic structure studies and in reconstructing phylogenetic relationships because of their simple structure, short length, fast evolutionary rate, and matrilineal inheritance [6,7,8]. In 1992, Liu et al. were the first to use the *cox2* gene of the mitochondrial genome to analyze 10 orders of insects phylogenetically, after which most studies on the mitochondrial genome of Lepidoptera involved only a selection of the genes, and studies using the whole mitochondrial genome were relatively few [9]. However, sequencing the entire mitochondrial genome and analyzing their structural features can help us understand the structural differences between different taxa at the molecular level and provide a reliable theoretical basis for morphological classification.

In this study, we sequenced, compared, analyzed, annotated, and assembled the mitochondrial genomes of 22 species of Sphingidae and reconstructed a phylogenetic tree. The phylogenetic relationships among the subfamilies of Sphingidae were analyzed to explore the origin, divergence time, and evolutionary status of diurnal sphingids. The mitochondrial genome compositions of eight diurnal hawkmoths were compared in detail, and the origin and evolution of diurnal behavior of hawkmoth is discussed [10,11].

## 2. Material and Methods

### 2.1. Sampling and DNA Extraction

Twenty-two specimens of Sphingidae (Table S1) were collected in Anhui Province, China, among which were six diurnal species: *Macroglossum bombylans* Boisduval, 1875, and *Macroglossum fritzei* Rothschild & Jordan, 1903, from Mount Qingliangfeng; *Macroglossum pyrrhosticta* Butler, 1875, and *Neogurelca himachala* (Butler, 1876) from Mount Jiulongfeng; *Hemaris radians* (Walker, 1856) from Mount Tiantangzhai, and *Sataspes xylocoparis* Butler, 1875, from Mount Bagongshan. Prior to DNA extraction, one leg was removed from an adult moth (male/female) and preserved in 100% ethanol at −20 °C. Total genomic DNA was extracted using the cetyltrimethyl ammonium bromide (CTAB) method [12]. DNA concentration and quality were estimated using the microliter UV/Vis spectrophotometer (Thermo Fisher Scientific, Wilmington, DE, USA) and agarose gel electrophoresis analysis.

### 2.2. Sequencing and Assembly

After quantification of the extracted DNA, a whole genome shotgun (WGS) strategy was used to sequence the DNA using high-throughput sequencing on an Illumina Novaseq platform using 150 paired-ends (PE) sequencing [13,14]. Raw data were processed by AdapterRemoval Version 2 and filtered into high quality data before being analyzed by single nucleotide polymorphism (SNP), insertion and deletion (InDel), copy number variation (CNV), and structural variation (SV) to ensure nucleotide reliability [15]. Assembly of the mitochondrial genome was done with Novoplasty [16]. The complete mitogenomes will be uploaded to GenBank (Table S1).

### 2.3. Mitochondrial Genome Annotation and Analysis

Gene annotation of mitochondrial genome was performed using MitoZ and 13 protein-coding genes, and a further 22 tRNA genes were annotated [17]. The complete mitochondrial genome sequence obtained by annotation was uploaded to the MITOS web server, and analytical parameters were set using the invertebrate genetic code [18]. Protein-coding genes were identified as open reading frames corresponding to the 13 PCGs of invertebrate animals. The rRNA genes and Control Regions (CR) were identified by the boundaries of the tRNA genes. Geneious Version 11.0.2 was used to examine all genes in the mitochondrial genome [19]. Mitogenomic maps were generated using the online OGDRAW Version 1.3.1 software [20].

MEGA Version 7.0 software was used to compute the numbers of non-synonymous and synonymous substitutions and nucleotide composition of the PCGs [21]. Composition skew values were calculated according to the formulae: GC − skew = (G − C)/(G + C) and ATskew = (A − T)/(A + T). Relative synonymous codon usage (RSCU) was calculated to determine whether there is codon usage bias in the coding sequences of the eight species. Tandem repeats in the CR were detected on the Tandem Repeats Finder web server, which produces results selected on the basis of copy number [22,23].

### 2.4. Phylogenetic Analysis

Using both maximum likelihood (ML) [24] and Bayesian inference (BI) methods [25], we constructed a phylogenetic tree of the 22 mitogenomes newly sequenced here, together with 33 sphingid and 17 Saturniidae mitogenomes downloaded from GenBank (Table S1), the latter used as the outgroup.

Multiple sequence alignment of protein-coding genes was performed using MAFFT 7 through the MAFFT webserver (https://mafft.cbrc.jp/alignment/server/, accessed on 10 May 2022) with the G-INS-I algorithm [26]. Alignments of individual genes were then concatenated into a combined matrix with DAMBE 5.3.74 [27]. Alignments of individual genes were concatenated to generate two data sets: (1) the PCGR matrix, including all three codon positions of protein-coding genes and two rRNA genes; (2) the PCG12 matrix, including only the first and second codon positions of protein-coding genes. The best-fit substitution model was automatically selected for ML phylogenetic trees using IQ-TREE Version 1.3.11, and branch supports were tested using ultrafast bootstrap and SH-aLRT tests (1000 replicates) [28,29]. BI analysis was performed using Phylobayes. Two million generations were run simultaneously, and trees were sampled every 100 generations. Once the Maxdiff value was less than 0.3 and the Effsize value was greater than 50, stationarity was considered to have been reached. The first 25% of the trees were then discarded as burn-in and the remaining trees used to generate a 50% consensus tree, which was then viewed using FigTree Version 1.4.4 [30].

### 2.5. Ancestral Character State Reconstruction

The 22 newly generated mitogenomes and 50 downloaded from GenBank were also analyzed to reconstruct the ancestral character states of adult lifestyle in Sphingidae. Lifestyle states were defined as follows: 1: diurnal behavior; 2: nocturnal behavior; 3: both diurnal and nocturnal behavior. The character matrix was analyzed using Mesquite 3.04 under a parsimony model. The results were edited using Adobe Illustrator CC 2018.

### 2.6. Divergence Time Estimation

Following Kawahara et al.’s research, the minimum and maximum bounds for two selected nodes were set as follows: (1) root: 48.8–69.0 Mya; (2) crown (Sphingidae): 29.6–44.9 Mya [31]. Using BEAST 1.8.3, the divergence time of Sphingidae was estimated using an uncorrelated lognormal relaxed molecular clock and Yule process and a run of 50 million generations, with trees sampled every 5000 generations [32]. The steady state and convergence were checked using Tracer 1.6 to ensure that the effective sampling size of all parameters was greater than 200. TreeAnnotator 1.8.3 was used to discard the first 25% of trees and to obtain node ages and 95% confidence intervals. FigTree Version 1.4.4 was used to view the generated tree.

## 3. Results

### 3.1. Genome Structure and Organization

The complete mitogenomes of all eight diurnal hawkmoths were found to be typical circular double-stranded molecules with the following lengths: *Cephonodes hylas*, 15,410 bp; *Hemaris radians*, 15,436 bp; *Macroglossum bombylans*, 15,461 bp; *M. fritzei*, 15,336 bp; *M. pyrrhosticta*, 15,348 bp; *M. stellatarum*, 15,290 bp; *Neogurelca himachala*, 15,264 bp; and *Sataspes xylocoparis*, 15,201 bp (Table S2). All the usual animal mitochondrial genes (13 PCGs, 22 tRNAs, and two rRNAs) and Control Regions (CRs) were identified. Twenty-three genes (14 tRNAs and nine PCGs) were transcribed from the majority strand (J strand), and the remaining 14 genes (four PCGs, two rRNAs, and eight tRNAs) were transcribed from the minority strand (N strand). The gene orders of the six newly sequenced diurnal hawkmoths were found to be identical to those of the two other diurnal species of Sphingidae that had been previously sequenced [10,33].

**Figure 1:**
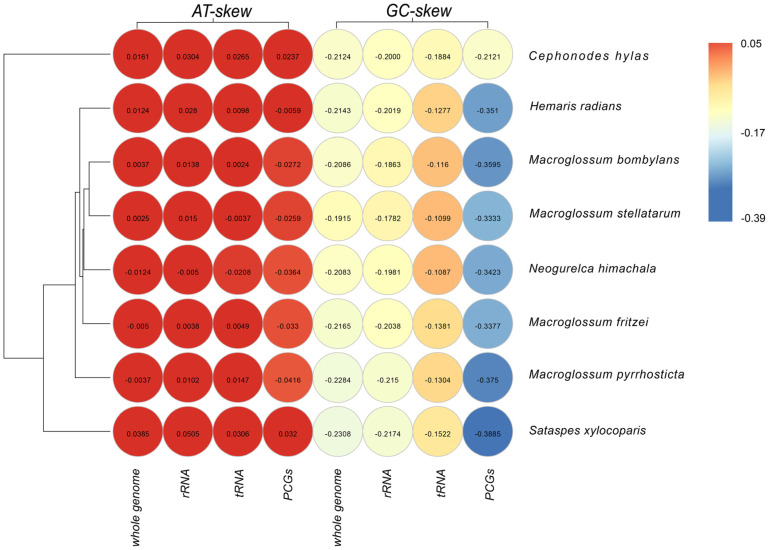
Nucleotide composition of various datasets of mitogenomes. Hierarchical clustering of eight diurnal hawkmoths based on the AT−skew and GC−skew.

The nucleotide compositions were investigated by calculating the percentages of AT-skew and GC-skew (Figure 1). The results showed that the AT-skews in the PCGs and tRNAs were almost all positive, while the GC-skews were almost all negative. Notably, the AT-skews in the rRNAs were almost all negative, due to the T-skews in the rRNAs being very high and exceeding those of A. The nucleotide composition of all the mitogenomes had a high A + T content, with an average of 80.73%, showing a strong A/T bias (Table S2).

### 3.2. Protein-Coding Genes (PCGs)

In the eight diurnal sphingid mitochondrial genomes, 9 of the 13 protein-coding genes were encoded by the majority strand, and 4 (*nad1*, *nad4*, *nad4l*, and *nad5*) were encoded by the minority strand. PCG lengths were 11,190 bp for *C. hylas* and *S. xylocoparis*, 11,193 bp for *H. radians*, *M. pyrrhosticta*, *M. stellatarum*, and *M. fritzei*, 11196bp for *M. bombylans*, and 11,212 bp for *N. himachala*, accounting for 72.62%, 73.61%, 72.51%, 72.93%, 73.20%, 72.99%, 72.41%, and 73.45% of their entire compositions, respectively.

In most insect mitochondrial genomes, the initiation codons are usually ATN [34]. However, all the *cox1* genes in these mitogenomes use CGA as the start codon. This phenomenon of non-standard start codons for *cox1* is also seen in other species [35,36]. The remaining 12 PCGs all use ATN as the initiation codon. Furthermore, three termination codons were found in the PCGs, namely TAA, TAG, and T (Table S3). In all mitogenomes, the occurrence frequency of the termination codon TAA was highest, and the termination codon T occurred least frequently. It is noteworthy that *cox1*, *cox2*, and *nad5* of these eight species all end in a single T. *nad3* of four species (*M. pyrrhosticta*, *M. stellatarum*, *N. himachala*, and *M. fritzei*), *nad1* of three species (*H. radians*, *M. fritzei*, and *S. xylocoparis*), and *nad4l* of two species (*M. bombylans and N. himachala*) all end with TAG. The remaining PCGs end with TAA. Thus, the frequently used codons consist exclusively of A and T, as in most insect mitochondrial genomes [37].

**Figure 2:**
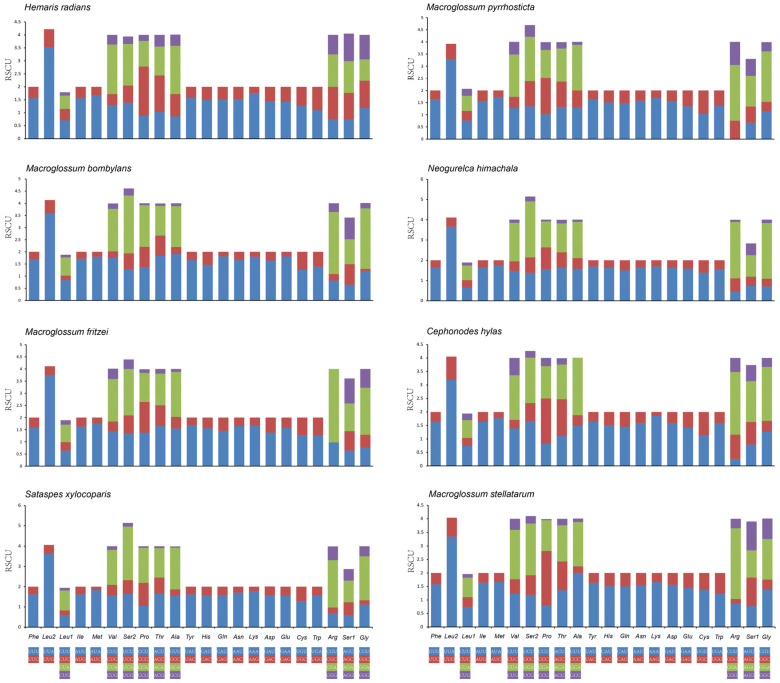
Relative synonymous codon usage (RSCU) of the mitochondrial genomes of eight diurnal hawkmoths. Codon families were provided on the *X*-axis. The value of the *Y*-axis indicates relative synonymous codon usage (RSCU).

Synonymous codons are generally used with different frequencies, a phenomenon known as codon bias [38]. Relative synonymous codon usage (RSCU) was calculated for the eight mitogenomes of diurnal hawkmoths (Figure 2). The amino acid compositions and RSCUs of these mitogenomes were similar. The most frequently used codons are UUA (Leu) and CGA (Arg), whereas the two most infrequently used codons are GCG (Ala) and CCG (Pro). The five most frequently used codons, UUA (Leu), AUU (Ile), UUU (Phe), AUA (Met), and AAU (Asn), all have high A + T content, which significantly increases A + T bias across the mitogenome.

**Figure 3:**
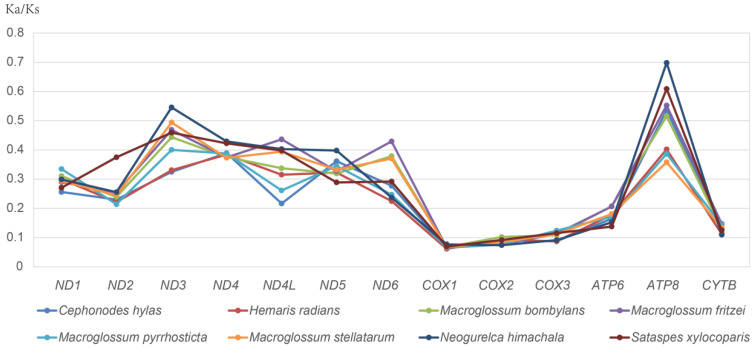
Evolutionary rates of 13 protein-coding genes of eight diurnal hawkmoths, comprising three cytochrome coxidase subunits (*cox 1-3*), cytochrome b (*cob*), two ATPase subunits (*atp6* and *atp8*), and seven nicotinamide adenine dehydrogenase subunits *(nad1-6* and *nad4L*). The numbers of nonsynonymous substitutions per nonsynonymous site (Ka), the number of substitutions per synonymous site (Ks), and the ratio of Ka/Ks for every genome are given, using *Neoris haraldi* as the reference sequence.

The ratio of non-synonymous substitutions per non-synonymous site (Ka) and the number of substitutions per synonymous site (Ks), Ka/Ks, provides information on sequence evolution. Increasing values of Ka/Ks imply positive selection, whereas decreasing values imply purifying selection [39]. In this study, we used *Neoris haraldi* Schawerda, 1923 (Saturniidae), as the reference sequence to calculate Ka/Ks ratios for each PCG (Figure 3). All values of Ka, Ks, and Ka/Ks were below 1, suggesting that the species are under purifying selection. The lowest evolutionary rate is *cox1*, and *nad3* and *atp8* have the higher sequence variability.

### 3.3. Transfer and Ribosomal RNA Genes

Two rRNA genes (12S and 16S rRNAs) were found on the minority strand in the eight mitogenomes. The 16S rRNA gene was 1280−1387 bp long and was found between *trnL1* and *trnV*, and the 12S rRNA, ranging from 766−812 bp in size, was located between the *trnV* and A + T-rich region. The shortest 12S rRNA and 16S rRNA are both in *M. stellatarum*. The average total size of the two rRNAs for these eight mitogenomes was 2140 bp, with an average A + T content of 84.7%.

The eight mitogenomes have the 22 usual tRNAs. The tRNA regions of the eight mitogenomes were 1468, 1470, 1471, 1453, 1458, 1464, 1454, and 1469 bp. Among the 22 tRNAs, eight (*trnQ*, *trnC*, *trnY*, *trnH*, *trnP*, *trnL1*, *trnV,* and *trnF*) were coded on the N strand and 14 on the J strand and range in size from 64 bp to 71 bp. The *trnS1* of all species lacked the dihydrouridine (DHU) arm but the other sequences can all be folded into the canonical cloverleaf secondary structure. A lack of the DHU arm in *trnS1* is found in the mitochondrial genomes of most insects and is considered a typical feature of insect mitogenomes [8]. Nucleotide substitutions and indels were found in the DHU loops and pseudouridine (TΨC) arms; for example, six such mismatches (U−G in *trnG*, *trnL2*, *trnP*, *trnQ*, *trnS1*, and *trnV*) were found in *H. radians*. It is noteworthy that two U−U mismatches occur in the anticcodon arm of *trnS2* for all species.

### 3.4. Intergenic Spacers and Overlapping Sequences

We observed 123 gaps in total in the eight mitochondrial genomes, with the sizes ranging from 1 to 59 bp (Table S3). The largest intergenic region (59 bp) is located between *nad2* and *trnQ* in *H. radians*. All eight species have one identical intergenic region in *nad2* and *trnQ*, which is consistent with previous studies [40]. Compared with the intergenic region, the number of overlapping gene regions is significantly reduced, and the overall length is also less. There are 50 overlapping regions of genes in the eight mitochondrial genomes, with a range of 1−17 bp. The longest overlap, 17 bp between *trnF* and *nad5,* was found in *H. radians*, *M. bombylans*, and *M. pyrrhosticta*. Additionally, the gene-overlap region between *trnW* and *trnC* (both 8 bp in length) is present in all eight mitochondrial genomes sequenced. *H. radians* had the most gaps (20) and *C. hylas* had the fewest (3).

### 3.5. Control Region

In the eight mitogenomes, the AT-rich region was found between *12S rRNA* and *trnM*. The CR of hawkmoths varies significantly between different species. In the eight diurnal hawkmoth mitogenomes, it ranges in size from 282 bp in *H. radians* to 507 bp in *M. bombylans*. Tandem repeats (TDRs) have frequently been reported in the mitochondrial AT-rich region of insects. In the present study, six of the eight diurnal hawkmoths (*C. hylas*, *H. radians*, *M. bombylans*, *M. pyrrhosticta*, *M. fritzei*, and *M. stellatarum*) contained tandem repeats in the CR, ranging in size from 14 to 44 bp. The length and copy number of the repeat units varies among Sphingidae species (Table S4). *Macroglossum pyrrhosticta* has the fewest repetitive sequences (2), whereas *M. stellatarum* has six repeats. The CR of both *C. hylas* and *M. bombylans* contains three tandem repeats, whereas those of *H. radians* and *M. fritzei* have four.

### 3.6. Phylogenetic Analyses

**Figure 4:**
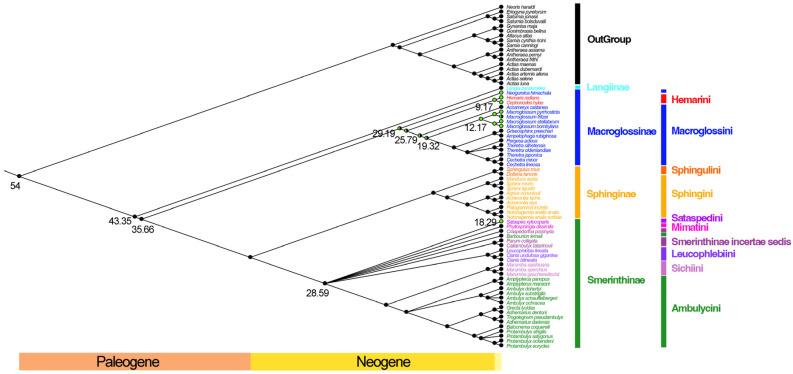
Ancestral state reconstruction on the phylogenetic tree based on PCG12 dataset. The number on the node indicates the estimated time of differentiation. Adult lifestyle was defined and is indicated as follows: (1) diurnal behavior (green dots); (2) nocturnal behavior (black dots); (3) both diurnal and nocturnal behavior (half black half green dots).

The phylogenetic relationships of Sphingidae reconstructed using Maximum Likelihood and Bayesian Inference were generally similar. Base composition heterogeneity and inter-site rate variation can affect phylogenetic inference [41] and removing third codon positions from protein-coding genes (PCGs) and RNAs (rRNAs and tRNAs) can reduce the effects of compositional heterogeneity, which is a necessary strategy for better resolving the family-level phylogeny [42,43]. Therefore, we consider that the PCG12 dataset is the most reliable and so only show the Ancestral state reconstruction on the phylogenetic tree based on PCG12 dataset (Figure 4).

The four currently recognized subfamilies (Langiinae, Macroglossinae, Sphinginae, and Smerinthinae), as proposed by Kitching [44], are all recovered as monophyletic with high support. Langiinae is the earliest differentiated group and sister to all remaining Sphingidae. This accords with the results of Kawahara [45], which was based on a small number of nuclear genes. Macroglossinae is the next clade to diverge as sister to a clade formed of Sphinginae + Smerinthinae. Of the eight diurnal hawkmoth species, one, *S. xylocoparis*, is placed within subfamily Smerinthinae as sister to the genus *Leucophlebia* Westwood, 1847, and the remaining seven species fall within subfamily Macroglossinae (Figure 4). *Neogurelca* is the first genus to branch off within Macroglossinae, followed by *Cephonodes* and *Hemaris*, which form a monophyletic tribe Hemarini (and thereby rendering tribe Macroglossini paraphyletic). Next to branch off is the nocturnal genus *Acosmeryx*, followed by the monophyletic diurnal genus *Macroglossum*. The remaining macroglossine taxa, *Griseophinx*−*Cechetra* are all nocturnal.

### 3.7. Divergence Time Estimation and Reconstruction of the Origin of Diurnalism

The ancestral state reconstruction of diurnalism (Figure 4) shows that Sphingidae first arose 43.35 million years ago (95% confidence interval: 33.65–46.7), when it split from its sister family, Saturniidae. Then, approximately in the Lutetian period of the Eocene, Sphingidae differentiated into Langiinae and the ancestor of the other three subfamilies at 43.35 Mya. The common ancestors of Macroglossinae and Sphinginae + Smerinthinae then differentiated 35.66 Mya (95% confidence interval: 29.03–42.19) during the Priabonian period of the late Eocene.

Among Macroglossinae, *N. himachala* was the first diurnal hawkmoth to diverge, originating at 29.19 Mya (95% confidence interval: 22.58–36.1). It was followed by *C. hylas* and *H. radians*, originating at 9.17 Mya (95% confidence interval: 4.48–17.45). Finally, four species in the Macroglossini, *M. bombylans*, *M. pyrrhosticta*, *M. stellatarum*, and *M. fritzei*, have the most recent common ancestor, originating at 12.17 Mya (95% confidence interval: 8.73–17.5). In Smerinthinae, only *S. xylocoparis* has diurnal behavior, which originated in 18.29 Mya (95% confidence interval: 10.46–25.5).

## 4. Discussion

In this paper, we describe the mitochondrial genomes of *C. hylas*, *H. radians*, *M. bombylans*, *M. pyrrhosticta*, *M. stellatarum*, *M. fritzei*, *N. himachala*, and *S. xylocoparis*. Of these, *C. hylas*, *H. radians*, *M. bombylans*, *M. pyrrhosticta*, *M. stellatarum*, *N. himachala*, and *S. xylocoparis* exhibit exclusively diurnal adult behavior, and *M. fritzei* combines both diurnal and nocturnal behavior. Taxonomically, *S. xylocoparis* belongs to subfamily Smerinthinae and the remainder to subfamily Macroglossinae.

The gene arrangement of the eight mitochondrial genomes is conserved and the sequence of genes is consistent. The size of the mitogenome varies among the examined species, ranging from 15,201 bp in *S. xylocoparis* to 15,461 bp in *M. bombylans*. These length differences are due mainly to variations in the length of the CRs and to random insertions in intergenic regions [46]. It is widely considered that due to a lack of the usual coding constraints, mitochondrial CRs have evolved faster than the protein-coding genes [47,48], and so mitochondrial CRs have been widely used to infer intraspecific and interspecific phylogenetic relationships.

In this study, six of the eight diurnal hawkmoth mitogenomes contained tandem repeat sequences in the CR but *N. himachala* and *S. xylocoparis* lacked them. However, *N. himachala* and *S. xylocoparis* are only quite distantly related, in different subfamilies, and the former is much closer to the remaining six diurnal species (Figure 4) that do have such duplicate units, and so their diurnal behavior is more likely to be the result of convergent evolution than to incomplete lineage sorting. The number and composition of the repeat units in different CRs are also different. *Macroglossum pyrrhosticta* has only two repeated sequences, but *M. stellatarum* has up to six. The repetitive sequence “TTAATTAAATAT” was found in the diurnal/nocturnal hawkmoth, *M. fritzei*. It is noteworthy that all diurnal hawkmoths with repetitive sequences in the CR species have the above sequence, but their repeats are longer than this segment, indicating that their repetitive sequences contain “TTAATTAAATATAT” within longer sequences.

Codon usage bias can be an indicator of the selective pressure operating at the molecular level [49]. The relative synonymous codon usage (RSCU) and codon distribution of the eight mitochondrial genomes (Figure 3) showed that the most commonly used codons are UUA (Leu2), CGA (Arg), GUU (Val), GCU (Ala), UCU (Ser2), and AGA (Ser1). In addition, the codons were biased to utilize more A/U than G/C, which resulted in the AT content being higher than GC in the PCGs. Further, among the eight mitogenomes, the most frequent initiation codon is ATN, whereas all *cox1* start with CGA. Frequent use of different start codons may reflect different evolutionary processes of mitochondrial genes in these diurnal hawkmoths compared with those in other hawkmoths. Regarding termination codons, all the PCGs end with TAA or TAG, while *cox2* ends with the incomplete termination codon T.

We estimated the average Ka/Ks values for each PCG to better understand the function of selection pressure and the development of the eight mitochondrial genomes. All PCGs had Ka/Ks values less than 1 (between 0.06 and 0.69), indicating that they are evolving under purifying selection. Nonsynonymous substitution occurs at a significantly lower rate than synonymous substitution because alterations in coding regions are frequently limited due to the impact they may have on protein function [50]. Among the 13 PCGs, *atp8* had the highest average Ka/Ks (0.51) and *cox1* (0.07) had the lowest. Among the eight hawkmoths, the mitochondrial protein-coding genes of *N. himachala* exhibited slightly higher Ka/Ks values than the other diurnal hawkmoths. It is worth noting that the Ka/Ks value of *nad2* in *S. xylocoparis* is 0.37, whereas the values for the other species are all less than 0.3. The conserved mitochondrial protein-coding sequences of *S. xylocoparis* may have been subjected to severe purifying selection to eliminate harmful mutations.

With regard to the adult flight period of the eight species that are the main subjects of the present study, *C. hylas*, *H. radians*, *M. bombylans*, *M. pyrrhosticta*, *M. stellatarum*, *N. himachala*, and *S. xylocoparis* all have exclusively diurnal behavior, whereas *M. fritzei* combines both diurnal and nocturnal behavior. According to the results of the ancestral trait reconstruction (Figure 4), diurnal behavior in Sphingidae arose twice among the species studied here, once in subfamily Macroglossinae 29.16 Mya (95% confidence interval: 22.58–36.1) and again in the genus *Sataspes* 18.29 Mya (95% confidence interval: 10.46–25.5). This may have led to the evolution of daytime flowering in some plant groups that had previously mostly flowered at night, and this facilitated the evolution of diurnalism in hawkmoths. At the same time, the Indian Plate continued to extrude northward and continuously insert under the Asian plate, causing the gradual uplift of the Tibetan Plateau, while gradually producing the Tibet-Himalayan orogenic belt, so differentiation of diurnal hawkmoths may also have been influenced by the uplift of the Tibetan Plateau and the surrounding region [51,52,53]. Alternatively, the main natural predators of nocturnal hawkmoths are bats, which originated in the Eocene (33.9–56.0 million years ago) and evolved on a large scale in the Oligocene-Pre-Miocene (20.44–33.9 million years ago). Many nocturnal hawkmoths can detect the echolocation signals used by bats to locate and track their prey through tympanic structures, thus avoiding predation to some extent, but those hawkmoths that do not possess this function may have been forced by predation pressure to switch to diurnal flight.

Mitochondrial DNA is an important source of genetic data and its limitations for the study of phylogenetic relationships have been well explored. It has been noted [54] that the mitogenome can be inadequate for resolving relationships at the subfamily level but has greater potential at lower levels, particularly if well sampled taxonomic units are available. Thus, our divergent dating estimates based on a phylogenetic tree constructed from the mitochondrial genome should only be considered as a provisional result. Furthermore, phylogenies based on mitochondrial genomes may conflict with those derived from nuclear genes, and the reasons for this need to be investigated. In the future, we hope to use genomic data and denser taxon sampling to reconstruct more robust phylogenetic relationships of hawkmoths and their relatives.

## Data Availability

The data that support the findings of this study are openly available in GenBank at https://www.ncbi.nlm.nih.gov/genbank/ (accessed on 3 August 2022). The list of investigated species and their Genbank accession numbers are in Table S1.

## Supplementary Materials

The following supporting information can be downloaded at: https://www.mdpi.com/article/10.3390/insects13100887/s1, Figure S1: Phylogenetic tree inferred from Bayesian Inference (BI) method based on PCG12 dataset. Numbers on branches are posterior probability values; Figure S2: Phylogenetic tree inferred from Maximum Likelihood (ML) method based on PCG12 dataset. Numbers on branches are bootstrap support values; Table S1. List of species investigated and their Genbank accession numbers. The GenBank numbers marked with an asterisk are the newly sequenced species in this paper; Table S2. Nucleotide composition and skewing of eight diurnal hawkmoths; Table S3. Mitogenomic organization of eight diurnal hawkmoths; Table S4. The tandem repeat units in the control region of the six diurnal hawkmoths.
