# Supplementary material for: Characterization of the Complete Mitochondrial Genome of Eight Diurnal Hawkmoths (Lepidoptera: Sphingidae): New Insights into the Origin and Evolution of Diurnalism in Sphingids
Source: Insects. 2022 Sep 29;13(10):887. doi: 10.3390/insects13100887 (PMC9604448; doi:10.3390/insects13100887)
Supplement: Supplementary file 1 [file insects-13-00887-s001.zip › insects-1875661-supplementary.pdf]

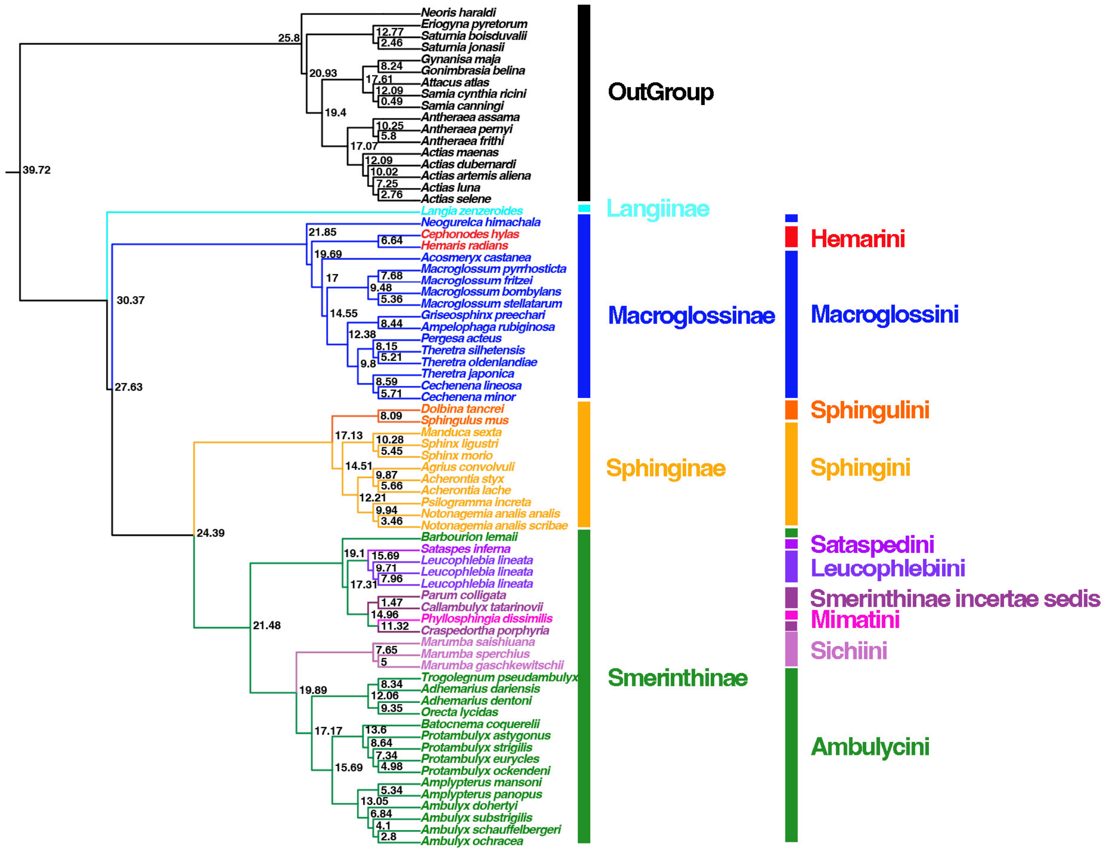

Figure S1. Phylogenetic tree inferred from Bayesian Inference (BI) method based on PCG12 dataset. Numbers on branches are posterior probability values.

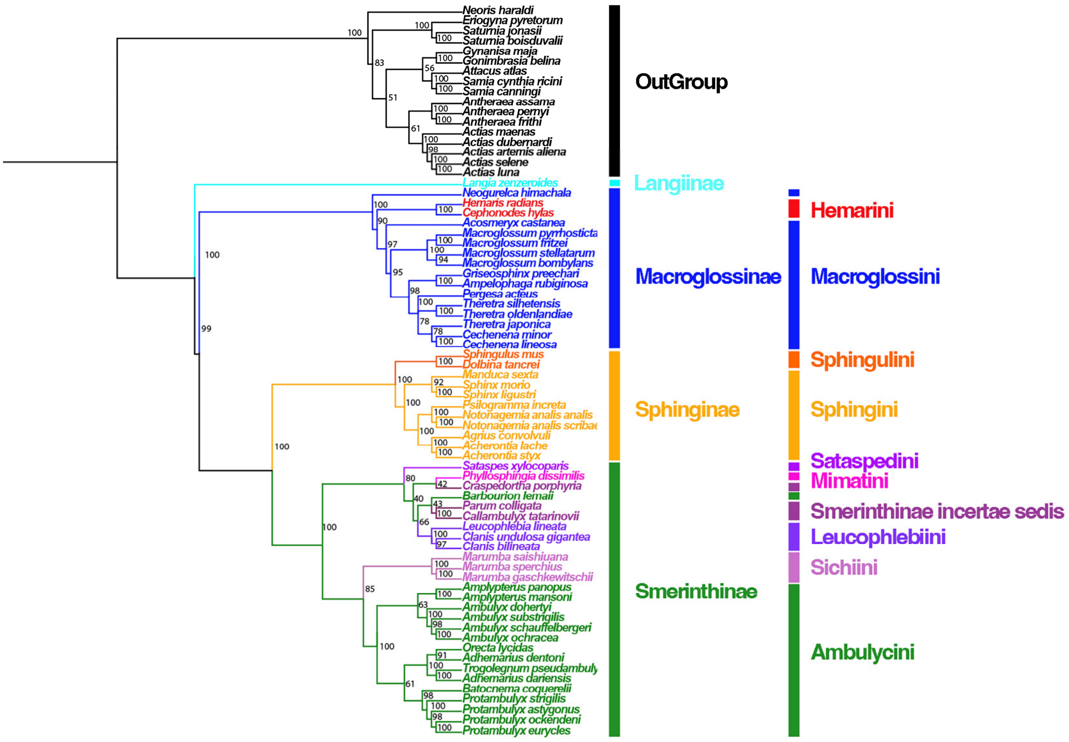

Figure S2. Phylogenetic tree inferred from Maximum Likelihood (ML) method based on PCG12 dataset. Numbers on branches are bootstrap support values.

Table S1. List of species investigated and their Genbank accession numbers. The GenBank numbers marked with an asterisk are the newly sequenced species in this paper.

NO.	Family	Taxa	GenBank Accession
1.	Saturniidae	*Actias artemis aliena*	KF927042
2.		*Actias dubernardi*	MW133617
3.		*Actias luna*	NC045899
4.		*Actias maenas*	MG836834
5.		*Actias selene*	JX186589
6.		*Antheraea assama*	NC030270
7.		*Antheraea frithi*	KJ740437
8.		*Antheraea pernyi*	MT890592
9.		*Attacus atlas*	NC021770
10.		*Eriogyna pyretorum*	NC012727
11.		*Gonimbrasia belina*	NC046032
12.		*Gynanisa maja*	NC046033
13.		*Neoris haraldi*	NC036765
14.		*Samia canningi*	NC024270
15.		*Samia cynthia ricini*	NC017869
16.		*Saturnia boisduvalii*	MF034742
17.		*Saturnia jonasii*	MF346379
18.	Sphingidae	*Acherontia lachesis*	OP219780*
19.		*Acherontia styx*	OP219781*
20.		*Acosmerycoides harterti*	MT712136
21.		*Acosmeryx castanea*	OP219769*
22.		*Adhemarius dariensis*	MK784108
23.		*Adhemarius dentoni*	MK804148
24.		*Agrius convolvuli*	OP219771*
25.		*Ambulyx dohertyi*	MK804150
26.		*Ambulyx ochracea*	MT712132
27.		*Ambulyx schauffelbergeri*	OP219770*
28.		*Ambulyx substrigilis*	MK804151
29.		*Ampelophaga rubiginosa*	MT712133
30.		*Amplypterus mansoni*	MK804152
31.		*Amplypterus panopus*	MK804153

NO.	Family	Taxa	GenBank Accession
32.		*Barbourion lemaii*	MK804154
33.		*Batocnema coquerelii*	MK804155
34.		*Callambulyx tatarinovii*	OP219772*
35.		*Cechenena lineosa*	OP219773*
36.		*Cechenena minor*	OP219774*
37.		*Cephonodes hylas*	MT712134
38.		*Clanis biilineata*	MK804156
39.		*Clanis undulosa gigantea*	MT712135
40.		*Craspedortha porphyria*	OP219775*
41.		*Dolbina tancrei*	OP219782*
42.		*Hemaris radians*	MZ593599*
43.		*Langia zenzeroides*	MT922035
44.		*Leucophlebia lineata*	MK804158
45.		*Macroglossum bombylans*	OP210702*
46.		*Macroglossum fritzei*	OP210703*
47.		*Macroglossum pyrrhosticta*	OP210704*
48.		*Macroglossum stellatarum*	MG747645
49.		*Manduca sexta*	EU286785
50.		*Marumba gaschkewitschii*	MT712137
51.		*Marumba saishiuana*	OP219776*
52.		*Marumba sperchius*	MT712138
53.		*Neogurelca himachala*	OP210705*
54.		*Notonagemia analis analis*	MT712143
55.		*Notonagemia analis scriba*	KU934302
56.		*Orecta Lycidas*	MK804159
57.		*Parum colligata*	MG888667
58.		*Pergesa acteus*	OP219777*
59.		*Phyllosphingia dissimilis sinensis*	OP219778*
60.		*Protambulyx astygonus*	NC046723
61.		*Protambulyx eurycles*	MK804161
62.		*Protambulyx ockendeni*	NC046725
63.		*Protambulyx strigilis*	MK804163
64.		*Psilogramma increta*	MF974243

NO.	Family	Taxa	GenBank Accession
65.		*Sataspes xylocoparis*	OP210706*
66.		*Sphingulus mus*	OP219783*
67.		*Sphinx ligustri*	OP219783*
68.		*Sphinx morio*	KC470083
69.		*Theretra japonica*	MG655620
70.		*Theretra oldenlandiae*	MN885801
71.		*Theretra silhetensis*	OP219783*
72.		*Trogolegnum pseudambulyx*	MK804164

Table S2. Nucleotide composition and skewing of eight diurnal hawkmoths.

	size	A%	C%	G%	T%	AT-skew	GC-skew
Cephonodes hylas	15410	41.00	11.70	7.60	39.70	0.0161	-0.2124
Hemaris radians	15436	40.70	11.90	7.70	39.70	0.0124	-0.2143
Macroglossum bombylans	15461	40.80	11.30	7.40	40.50	0.0037	-0.2086
Macroglossum fritzei	15336	40.10	11.80	7.60	40.50	-0.0050	-0.2165
Macroglossum pyrrhosticta	15348	40.00	12.10	7.60	40.30	-0.0037	-0.2284
Macroglossum stellatarum	15290	40.70	11.20	7.60	40.50	0.0025	-0.1915
Neogurelca himachala	15264	39.90	11.60	7.60	40.90	-0.0124	-0.2083
Sataspes xylocoparis	15201	41.80	12.00	7.50	38.70	0.0385	-0.2307

Table S3. Mitogenomic organization of eight diurnal hawkmoths.

	Position		Size (bp)	Intergenic nucleotides	Codon		Strand
	From	To			Start	Stop	
M. pyrrhosticta/ M. stellatarum/ N. himachala/ S. xylocoparis							
trnM	1/1/1/1	67/69/67/68	67/69/67/68				+/+/+/+
trnI	74/73/73/75	137/136/136/139	64/64/64/65	6/3/5/6			+/+/+/+
trnQ	203/202/202/205	135/134/134/137	69/69/69/69	-3/-3/-3/-3			-/-/-/-
nad2	258/260/255/262	1271/1273/1265/1275	1014/1014/1011/1014	54/57/52/56	ATT/ATT/ATT/ATT	TAA/TAA/TAA/TAA	-/-/-/-
trnW	1270/1273/1265/1281	1336/1343/1333/1347	67/71/69/67	-2/-/-/5			+/+/+/+
trnC	1392/1399/1391/1403	1329/1336/1326/1340	64/64/66/64	-8/-8/-8/-8			-/-/-/-
trnY	1458/1468/1458/1470	1393/1404/1394/1404	66/65/65/67	-/4/2/-			+/+/+/+
cox1	1466/1475/1462/1478	2996/3005/2992/3008	1531/1531/1531/1531	7/6/3/7	CGA/CGA/CGA/CGA	T/T/T/T	-/-/-/-
trnL2	2997/3006/2993/3009	3063/3072/3058/3075	67/67/66/67	-5/-/-/-			+/+/+/+
cox2	3064/3073/3059/3076	3745/3754/3740/3757	682/682/682/682	-/-/-/-	ATG/ATG/ATG/ATG	T/T/T/T	-/-/-/-
trnK	3746/3755/3741/3758	3816/3825/3811/3827	71/71/71/70	-/-/-/-			+/+/+/+
trnD	3830/3852/3839/3828	3895/3917/3903/3895	66/66/65/68	13/26/27/-			+/+/+/+
atp8	3896/3918/3904/3896	4063/4082/4077/4054	168/165/174/159	-/-/-/-	ATA/ATC/ATA/ATG	TAA/TAA/TAA/TAA	-/-/-/-
atp6	4057/4076/4071/4048	4734/4753/4748/4725	678/678/678/678	-7/-7/-7/-7	ATG/ATG/ATG/ATG	TAA/TAA/TAA/TAA	+/+/+/+
cox3	4734/4754/4748/4725	5525/5545/5539/5516	792/792/792/792	-/-/-/-	ATG/ATG/ATG/ATG	TAATAA/TAA/TAA	-/-/-/-
trnG	5528/5548/5542/5519	5593/5613/5607/5586	66/66/66/68	2/2/2/2			+/+/+/+
nad3	5594/5614/5608/5587	5947/5967/5961/5940	354/354/354/354	-/-/-/-	ATT/ATT/ATT/ATT	TAG/TAG/TAG/TAA	-/-/-/-
trnA	5946/5966/5960/5943	6016/6035/6024/6011	71/70/65/69	-2/-2/-/2			+/+/+/+
trnR	6017/6035/6025/6012	6081/6099/6088/6079	65/65/64/68	-/-/-/-			+/+/+/+
trnN	6090/6104/6091/6080	6155/6170/6156/6144	66/67/66/65	8/4/2/-			+/+/+/+
trnS1	6151/6171/6162/6145	6219/6236/6227/6208	64/66/66/64	-/-/5/-			+/+/+/+
trnE	6235/6237/6239/6211	6299/6301/6304/6276	65/65/66/66	15/-/11/2			+/+/+/+
trnF	6361/6365/6367/6343	6298/6300/6333/6275	64/66/65/69	-2/-2/-2/-2			+/+/+/+
nad5	8099/8103/8110/8078	6362/6366/6368/6344	1738/1738/1741/1735	-/-/-/-	ATT/ATT/ATT/ATT	T/T/T/T	-/-/-/-

trnH	8165/8168/8174/8145	8100/8104/8111/8079	66/65/64/64	15/-/-/-			+/+/+/+
nad4	9501/9508/9511/9482	8167/8174/8174/8145	1335/1335/1338/1338	1/5/-/-	ATG/ATG/ATG/ATG	TAA/TAA/TAA/TAA	-/-/-/-
nad4l	9793/9806/9809/9778	9503/9516/9522/9488	291/291/288/291	1/7/10/5	ATG/ATG/ATG/ATG	TAA/TAA/TAG/TAA	-/+/-/-
trnT	9798/9813/9815/9785	9862/9877/9880/9850	65/65/66/66	4/6/5/6			+/+/+/+
trnP	9927/9971/9945/9914	9863/9877/9881/9850	65/65/65/65	-/-/-/-			-/-/-/-
nad6	9932/9945/9950/9922	10456/10472/10480/10449	525/528/531/528	4/3/4/7	ATT/ATT/ATT/ATT	TAA/TAA/TAA/TAA	-/-/-/-
cytb	10456/10473/10480/10449	11604/11621/11634/11600	1149/1149/1155/1152	-/-/-/-	ATG/ATG/ATG/ATG	TAA/TAA/TAA/TAA	+/-/+/+
trnS2	11603/11624/11634/11599	11669/11688/11700/11664	67/65/67/66	-2/2/-/-2			+/+/+/+
nad1	12624/12643/12659/12622	11689/11708/11723/11687	936/936/937/936	19/19/22/22	ATG/ATG/ATG/ATG	TAA/TAA/TAA/TAG	-/-/-/-
trnL1	12693/12712/12728/12690	12626/12645/12661/12624	68/68/68/67	1/1/1/1			+/+/+/+
rrnL	14050/14000/14100/14039	12694/12721/12729/12691	1357/1280/1372/1349	-/-/-/-			-/-/-/-
trnV	14115/14130/14164/14103	14051/14066/14101/14040	65/65/64/64	-/65/-/-			+/+/+/+
rrnS	14922/14896/14941/14883	14116/14131/14165/14104	807/766/777/780	-/-/-/-			-/-/-/-
AT-rich	14923/14897/14942/14884	15348/15290/15264/15201	426/394/323/318	-/-/-/-			-/-/-/-

Table S4. The tandem repeat units in the control region of the six diurnal hawkmoths.

	Number of Repeats	Indices	Period Size	Copy Number
Cephonodes hylas	3	130-260	46	2.9
		152-244	36	2.5
		197-270	16	4.5
Hemaris radians	6	100-161	23	2.7
		102-157	19	3
		99-244	10	14.1
		167-237	2	38.5
		172-237	16	4.3
		157-232	40	1.9
Macroglossum bombylans	5	43-76	2	17
		228-291	26	2.6
		230-303	18	4.1
		53-450	208	1.9
		384-456	25	3.1
Macroglossum fritzei	6	13-82	2	36.5
		20-106	8	9.3
		173-247	25	3
		192-301	9	12
		259-312	12	4.4
		311-361	2	26
Macroglossum pyrrhosticta	3	28-65	1	38
		83-129	8	5.5
		286-334	20	2.3
Macroglossum stellatarum	7	53-120	2	37
		53-103	18	2.9
		54-130	26	2.9
		54-130	14	5.3
		53-137	28	3.1
		254-327	18	4.1
		233-353	28	4.3
